# Untargeted Assessment of Tumor Fractions in Plasma for Monitoring and Prognostication from Metastatic Breast Cancer Patients Undergoing Systemic Treatment

**DOI:** 10.3390/cancers11081171

**Published:** 2019-08-14

**Authors:** Christoph Suppan, Iva Brcic, Verena Tiran, Hannah D Mueller, Florian Posch, Martina Auer, Erkan Ercan, Peter Ulz, Richard J Cote, Ram H Datar, Nadia Dandachi, Ellen Heitzer, Marija Balic

**Affiliations:** 1 Division of Oncology, Department of Internal Medicine, Medical University of Graz, 8036 Graz, Austria; 2 Institute of Pathology, Medical University of Graz, 8010 Graz, Austria; 3 Institute of Human Genetics, Diagnostic and Research Center for Molecular Biomedicine, Medical University of Graz, 8010 Graz, Austria; 4 Department of Pathology, University of Miami Miller School of Medicine, Miami, FL 33136, USA; 5 Research Unit Epigenetic and Genetic Cancer Biomarkers, Medical University of Graz, 8036 Graz, Austria; 6 BioTechMed-Graz, 8036 Graz, Austria; 7 Christian Doppler Laboratory for Liquid Biopsies for Early Detection of Cancer, 8010 Graz, Austria; 8 Research Unit Circulating Tumor Cells and Cancer Stem Cells, Medical University of Graz, 8036 Graz, Austria

**Keywords:** metastatic breast cancer (MBC), circulating tumor cells (CTCs), cell free circulating tumor DNA (ctDNA), mFAST-SeqS, prognosis, treatment response

## Abstract

The aim of this study was to assess the prognostic and predictive value of an untargeted assessment of tumor fractions in the plasma of metastatic breast cancer patients and to compare circulating tumor DNA (ctDNA) with circulating tumor cells (CTC) and conventional tumor markers. In metastatic breast cancer patients (*n* = 29), tumor fractions in plasma were assessed using the untargeted mFAST-SeqS method from 127 serial blood samples. Resulting z-scores for the ctDNA were compared to tumor fractions established with the recently published ichorCNA algorithm and associated with the clinical outcome. We observed a close correlation between mFAST-SeqS z-scores and ichorCNA ctDNA quantifications. Patients with mFAST-SeqS z-scores above three (34.5%) showed significantly worse overall survival (*p* = 0.014) and progression-free survival (*p* = 0.018) compared to patients with lower values. Elevated *z*-score values were clearly associated with radiologically proven progression. The baseline CTC count, carcinoembryonic antigen (CEA), and cancer antigen (CA)15-5 had no prognostic impact on the outcome of patients in the analyzed cohort. This proof of principle study demonstrates the prognostic impact of ctDNA levels detected with mFAST-SeqS as a very fast and cost-effective means to assess the ctDNA fraction without prior knowledge of the genetic landscape of the tumor. Furthermore, mFAST-SeqS-based ctDNA levels provided an early means of measuring treatment response.

## 1. Introduction

Breast cancer (BC) is one of the leading causes of cancer-related death in the global female population [1]. Despite advances in targeted and systemic treatment, metastatic disease remains incurable and lethal [2], and there is an unmet need to identify prognostic and predictive factors for early detection of disease progression and response to therapy. Although cancer antigen (CA) 15-3 and carcinoembryonic antigen (CEA) are widely used for surveillance purposes, their usage in screening or therapeutic response monitoring is not recommended due to limited sensitivity and specificity [3,4,5]. Other blood-based biomarkers such as circulating tumor cells (CTCs) and cell-free circulating tumor DNA (ctDNA) have been extensively studied, and numerous studies have demonstrated their prognostic and predictive value in several solid malignant tumors [6,7,8,9,10,11,12,13,14].

CTCs can be detected in up to 80% of metastatic breast cancer (MBC) patients [6,7,8,9] and are associated with poor progression-free survival (PFS) and overall survival (OS) [10,11,12,13,14,15]. In addition, reduced numbers of CTCs after systemic therapy were found to be an independent predictor of better prognosis [16,17]. As the molecular analysis of CTCs is challenging, many studies concentrated on the enumeration of CTCs. In contrast, ctDNA can be comprehensively analyzed with a variety of methods without the need for costly enrichment technologies [18]. Moreover, ctDNA represents bulk DNA from different tumor locations, providing a more comprehensive picture of the genetic landscape of the tumor [19,20]. For the assessment of the tumor load in the circulation, mostly known mutations from the primary tumor are tracked in plasma [21]. However, tumors are constantly evolving and in advanced stages, genetic alterations in metastases may significantly differ from those identified in the primary tumor [22].

Genome-wide, untargeted approaches do not require a priori knowledge about genetic alterations of the respective tumor. Such genome-wide approaches may consist of whole-genome sequencing (WGS) of plasma DNA [23,24,25,26]. We previously demonstrated that WGS with a shallow sequencing depth of 0.1–0.2x allows for reliable detections of somatic copy number alterations (SCNAs) from patients with cancer and termed this approach plasma-Seq [26,27,28,29,30]. Recently, such ultra-low-pass whole-genome sequencing (ULP-WGS) data were evaluated with a new algorithm called ichorCNA to estimate the tumor content in plasma DNA [31]. Genome-wide, untargeted approaches can also be amplicon-based. For example, mFAST-SeqS employs selective amplification of *LINE1*-sequences across the genome and allows detection of SCNAs at a chromosome-arm level, representing a fast and cost-effective untargeted assessment of tumor fractions [32]. However, due to limited analytical sensitivity, untargeted approaches can only be used in cases where tumor-derived DNA accounts for more than 3%–10% of the total circulating cell-free DNA (cfDNA).

In this proof-of-concept study, we tested both the prognostic value of the mFAST-SeqS method and its applicability as a monitoring tool for treatment response. Moreover, since little data are available on the combined analysis of ctDNA, CTCs and conventional protein tumor markers, we compared the performance of ctDNA, CTCs detected by size-based CTC microfilter enrichment and panCK to CD45 staining, CEA and CA15-3. Thus, we elucidated whether these circulating components provide similar or complementary information with respect to prognosis and monitoring treatment response in MBC patients.

## 2. Results

### 2.1. Study Population

At primary blood sampling, the median age of the study population (*n* = 29) was 56 years (25–75th percentile: 50–68). After a median follow-up time of 27 months (±1.9), disease progression was observed in 25/29 cases (86.2%), and death in 12/29 cases (41.4%). Overall, bone metastases were present in 20 (69.0%) patients, liver metastasis in 10 (34.5%), lung in 13 (44.8%) and other metastases in 22 patients (75.9%). All patients were treated by one or more systemic therapies, including chemotherapy, hormone therapy, immune therapy and combinations thereof. Baseline characteristics, including the number of previous treatments, are listed in Table 1.

### 2.2. Untargeted Assessment of Tumor Fraction

In the 29 patients, ctDNA was analyzed in a total of 127 serial blood samples, CTCs in 97 samples, and CEA and CA15-3 each in 112 blood samples. Data comparing ctDNA and CTC were available for 97 samples from 29 patients.

Tumor fractions were assessed with the previously described mFAST-SeqS assay [32]. Originally, the assay was developed to select plasma samples with high tumor fractions (>5%–10%) where higher-resolution genome-wide approaches such as plasma-Seq are applicable [26]. Here we established for the first time the correlation of the mFAST-SeqS *z*-score with the tumor fraction in plasma, and to this end, we applied the recently published ichorCNA algorithm, a tool for estimating the fraction of tumor-derived fragments in cfDNA of ULP-WGS [31], to a total of 72 selected samples with a wide range of *z*-scores (range −0.19–124.98). To this end, we performed ULP-WGS of all samples with elevated *z*-scores (*n* = 39) and of samples with *z*-scores below 3 (*n* = 33). As expected, samples with *z*-scores below 3 did not yield informative results due to low tumor fractions.

**Figure 1:**
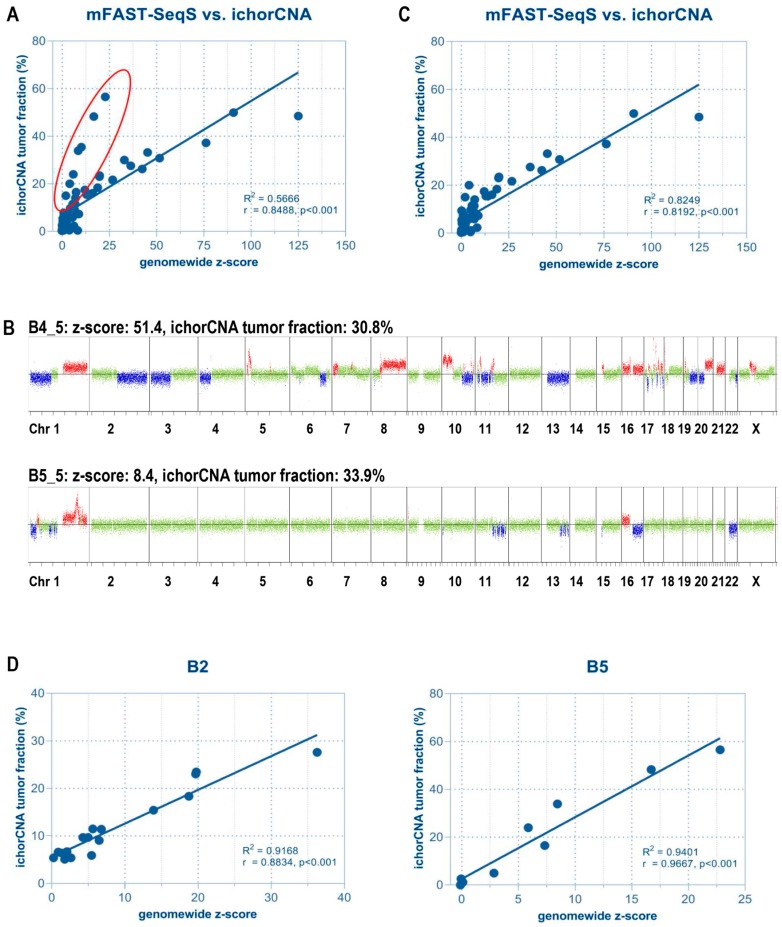
Correlation between mFast-SeqS based z-scores and ichorCNA tumor fractions. (A) With the exception of some samples (highlighted in red), the majority showed a high correlation between z-score and tumor fraction. Most of these outliers were obtained from patient B5. (B) Samples with high tumor fraction but lower z-scores revealed a lower number of copy number alterations (CNAs) compared to other samples as exemplified by two genome-wide copy number profiles. Based on ichorCNA, both samples had a tumor fraction of approximately 30%. However, due to the low abundance of CNAs in patient B5, the mFAST-SeqS z-score was only 8.4, compared to a value of 51.4 of a patient with many CNAs across the genome. Red indicates a gain of chromosomal material, blue indicates a loss of chromosomal material, green depicts balanced regions. (C) The removal of samples with low overall amounts of CNA significantly improved the correlation. (D) Correlation between mFAST-SeqS z-scores and ichorCNA-based tumor fractions in selected individual patients.

As expected, most samples with high *z*-scores showed elevated tumor fractions and vice versa (e.g., B4 in Figure 1B). In fact, we observed high correlation between ichorCNA and mFAST-SeqS for the entire cohort (*n* = 72, *R*^2^ = 0.5666, Spearman correlation r = 0.8488, *p* < 0.001) (Figure 1A). Interestingly, we observed a subset of samples with high tumor fractions according to ichorCNA but only moderately elevated *z*-scores (Figure 1A). These were samples where SCNAs involved only a few chromosome arms and which were derived mostly from patient B5 (Figure 1B). In such cases, mFAST-SeqS may underestimate the true ctDNA level as the genome-wide mFAST-SeqS *z*-score is calculated from all chromosome-arm specific *z*-scores. When omitting these samples from the analysis, mFAST-SeqS *z*-scores again correlated closely with the ichorCNA estimations (*n* = 61, *R*^2^ = 0.8299, r = 0.8192, *p* < 0.001) (Figure 1C). Moreover, as SCNAs do not change drastically from one analysis to the next (Figure S1), we reasoned that the correlation should be even higher for samples from individual patients. This was indeed the case (Figure 1D, Figure S2), suggesting that mFAST-SeqS *z*-scores should be excellently suited for disease monitoring.

## 3. Prognostic Impact of ctDNA, CTCs and Tumor Markers on Survival

**Figure 2:**
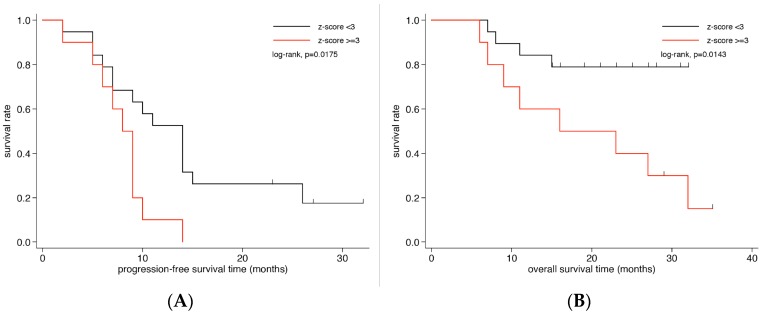
Kaplan–Meier survival analysis according to *z*-scores in metastatic breast cancer patients. Kaplan–Meier curves show that patients with *z*-scores ≥3 have significantly less (**B**) overall survival and (**A**) progression-free survival.

To evaluate whether the level of aneuploidy and/or high tumor fractions in plasma were associated with survival, we stratified patients according to their baseline *z*-scores. After testing several cut-offs (<2 vs. ≥2; <3 vs. ≥3; <4 vs. ≥4; <5 vs. ≥5), a *z*-score of 3 was identified as the value that best discriminated OS and PFS. The OS time of the 19 patients with a *z*-score below 3 (median not reached) was significantly higher than that of the 10 patients with a *z*-score ≥ 3 (16.0 months; 95% CI 6–32 months; log-rank test, *p* = 0.014; Figure 2B). Kaplan–Meier estimates of OS of two years were 40% for patients with a *z*-score ≥ 3 and 79% for patients with a *z*-score < 3. Moreover, patients with *z*-scores below 3 demonstrated significantly longer PFS than those with elevated *z*-scores (14 (CI 7–15) vs. 8 (CI 2–9) months, *p* = 0.018, Figure 2A). In contrast, baseline CTC counts, CEA or CA15-3 were not associated with PFS or OS, respectively (Figure S3). While limited by small samples sizes, the *z*-score was not an independent prognostic factor in a multivariate model using a Cox proportional-hazard model including the *z*-score (<3 vs. ≥3) and the intrinsic tumor subtype.

## 4. Association of Tumor Fractions, CTC Detection Rate, Tumor Marker and Clinicopathological Characteristics

Elevated levels of CEA (>5 ng/mL) and CA15-3 (>31 U/mL) at baseline were observed in 14/27 (51.9%) and 19/27 (70.4%) patients, respectively. In contrast, a *z*-score of ≥3 at baseline was seen in only 10/29 patients (34.5%), while a total of 17 patients (58.6%) showed ≥ 1 CTC at baseline. CTCs (≥1) or *Z*-score (≥3) positivity was found in 19 patients (65.5%).

Data comparing ctDNA and CTC were available for 97 samples from 29 patients. 31/97 samples (32.0%) had a *z*-score above 3 and 49/97 (50.5%) samples were positive for at least one CTC. In 62/97 (63.9%) samples either a *z*-score above 3 or one or more CTCs were observed, whereas only 18/97 (18.6%) of analyzed samples showed both elevated ctDNA and CTCs. Data comparing ctDNA and tumor markers were available for 29 patients and 112 samples. Of these, 41 (36.6%) samples had a *z*-score above 3, 57 (50.9%) samples had elevated levels for CEA and 76 (67.9%) samples revealed elevated CA15-3 levels. Following from this, 25/112 (22.3%) samples had a *z*-score above 3 and elevated CEA levels, while 34 (30.4%) samples had a *z*-score above 3 and elevated CA15-3 levels.

Taken together, there was no quantitative relationship between the genome-wide mFAST-SeqS *z*-score and the number of CTCs. A significant but low positive correlation was found between ctDNA and tumor markers and between the CTC count and CA15-3 (Table 2). When we associated the tumor fraction and the CTC status at baseline with clinicopathological factors including menopausal status, histology, grading, tumor size, lymph node status, ER-/PR-status and bone, lung or liver metastasis, no significant correlation was observed except for CTC positivity and liver metastasis (Table 3).

## 5. Serial Monitoring of the Genome-Wide *z*-Score of ctDNA

We investigated whether quantitative changes in mFAST-SeqS *z*-scores during treatment reflect the response to therapy and disease progression. twenty-nine patients contributed 108 individual plasma samples (mean 3.7, range 1–8). The median time between first and last *z*-score measurement was 7 weeks (25th–75th percentile: 0–15.5). Using a linear mixed model with a random intercept at the patient level, the mean estimates for *z*-score at baseline was 8.21 (95% CI: 1.91–14.52), 2.81 (95% CI: −1.77–7.39) during treatment, and 17.56 (95% CI: 8.82–26.29) at disease progression (Wald test, *p* = 0.007). Specifically, we found that a higher *z*-score was significantly associated with disease progression compared to treatment *z*-scores (Wald test, *p* = 0.002), while no difference was found between mean baseline *z*-scores and mean treatment *z*-score (Wald test, *p* = 0.150). CA15-3 showed similar results, while CEA and CTCs did not show significant differences at these time points (Figure S4).

**Figure 3:**
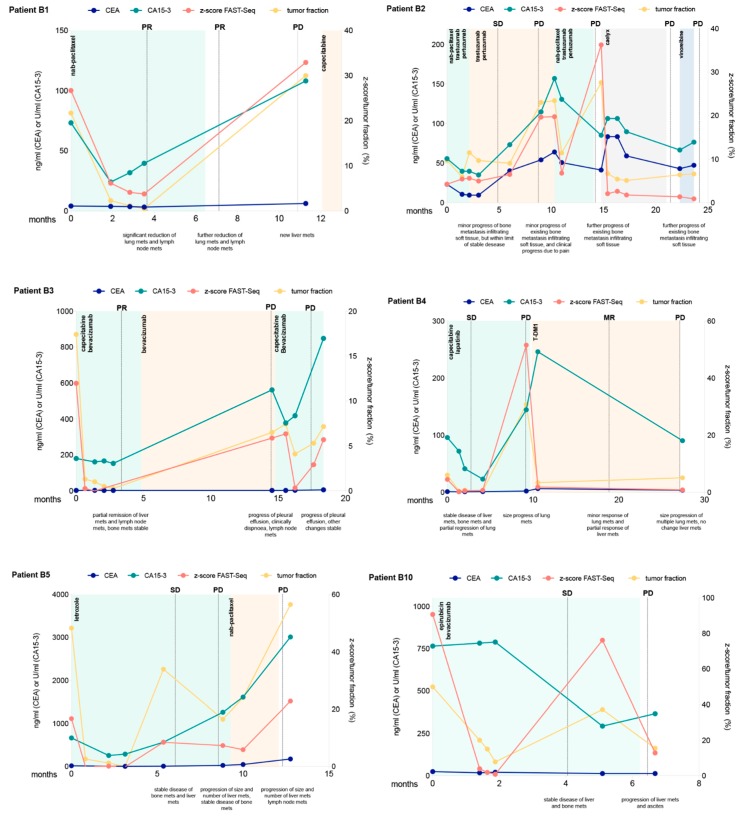

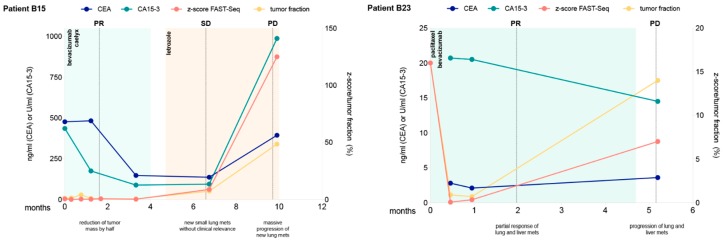
Longitudinal monitoring of *z*-scores and ichorCNA tumor fractions in circulating tumor DNA indicated for eight selected patients. Multiple measurements of tumor markers CEA and CA15-3 are also shown over time. Therapy regimens are indicated as colored shading and disease status at various times (ascertained on computed tomography or MRI) is shown as vertical dotted lines. SD denotes stable disease, PD progressive disease, PR partial response and MR minor response.

In 13 (46%) patients, changing levels of tumor fraction based on *z*-scores could be observed, while in 15 patients (54%), *z*-scores were-despite progressive disease-below 3 for all analyzed time points and therefore uninformative. However, for those patients including at least one sample with an elevated *z*-score, serial *z*-score assessment correlated with radiographic response to therapy (Figure 3 and Figure S5). In most of these patients, *z*-scores were above 3 at treatment initiation and decreased with a radiographic response. At the time of progression, or when a response could not be achieved, *z*-scores increased or remained elevated (Figure 3 and Figure S5). The same result was observed for ichorCNA tumor fractions. While CA15-3 was in most cases closely correlated to the ctDNA fraction and the *z*-score, CEA did not reflect the response.

## 6. Discussion

An association of tumor fraction in plasma and prognosis has already been observed in a variety of tumor entities [29,33,34,35,36]. Monitoring of ctDNA levels usually requires the identification of specific somatic alterations in individual patients. In many advanced breast cancer cases, metastatic tumor tissue is not available for analysis. The fact that the driver landscape of metastases differs from primary cancers [37] highlights the need for an untargeted assessment of the tumor load without a priori knowledge of specific changes of the tumor in the circulation. Here, we demonstrate that an untargeted assessment of tumor fraction using the mFAST-SeqS method adds significant information for prognosis and response to treatment in MBC patients. A *z*-score of 3 or higher at treatment initiation was clearly associated with diminished OS and PFS. In contrast, the number of CTCs and the levels of CA15-3 or CEA were not informative. The reasons why our study failed to confirm a prognostic role of CTCs in metastatic breast cancer patients as previously reported include small sample size, heterogeneity of our patient cohort and a different CTC enrichment and detection method. We are also aware that our CTC detection was based on CK expression, and thus we could have missed CTCs in EMT or with stem cell character. While at the current stage our data may be indicative that detection of CTCs based on microfilter enrichment was not of prognostic significance, we strongly believe that our study did not have sufficient power to evaluate this question, and in order to test this, a larger more homogenous trial should be performed. On the other hand, we could clearly show that *z*-score based DNA levels were a significant prognostic marker despite the mentioned limitations and we strongly believe that these data warrant further confirmative studies.

A similar prognostic value for mFAST-SeqS was obtained from other tumor entities, such as prostate cancer patients [29] and non-small cell lung cancer patients [38] indicating that elevated tumor fractions seem to be an independent negative prognostic factor in a variety of tumor entities. Recently, ichorCNA [31] was used to stratify 164 patients with metastatic triple-negative BC based on high and low tumor fractions (threshold) of ≥10%. Similarly to our data, high tumor fractions were associated with significantly decreased survival (median, 6.4 v 15.9 months) [33] confirming the strong prognostic effect of high tumor levels in the plasma of MBC patients. Comparisons of mFAST-SeqS *z*-scores with ichorCNA tumor fractions demonstrated a good correlation. Importantly, the assessment of quantitative changes of *z*-scores within samples from the same patient showed a strong correlation with tumor fractions, suggesting that *z*-scores present an excellent measure for the longitudinal assessment of changing levels of ctDNA in individual patients. We confirmed this by evaluating mFAST-SeqS as a monitoring tool. Indeed, elevated *z*-scores in patients with informative values were clearly associated with radiologically proven progression in most cases, while a response to treatment resulted in a significant decrease of *z*-scores. Several studies have shown similar results demonstrating that fluctuations of mutant allele fractions in plasma over time may show a relation to tumor response [19,39,40]. In contrast to previous data, neither changing numbers of CTCs nor changing levels of CEA reflected treatment response.

Limitations of our study include the small sample size and the heterogeneity of our patient cohort. However, an elevated tumor fraction does not appear to be restricted to subtypes, suggesting a greater release of tumor-associated DNA into circulation in patients with more aggressive disease. Compared to ichorCNA and other WGS approaches, mFAST-SeqS resolution is limited to SCNA calling at chromosome-arm levels, and SCNA identification depends on the presence of *LINE*1 sequences within the altered region. Another drawback of the method is its limited analytical sensitivity. Therefore, advanced stages, which usually have elevated ctDNA levels, will be the main area of application. Such advanced stages are, in fact, those at which most clinical trials are being conducted, and therefore our approach could evolve into an important companion diagnostic tool for these studies. Furthermore, an improvement of sensitivity can be achieved by increasing the sequencing depth and/or bioinformatic approaches. For example, recently, an improved *LINE1*-based method called Within-Sample AneupLoidy DetectiOn (WALDO), which employs supervised machine learning to detect allelic imbalances and microsatellite instability down to of 1% neoplastic content, was reported [41].

Nevertheless, mFAST-SeqS offers three important advantages over other ctDNA analysis methods: (i) Speed: As it is based on a two-step PCR, it can be performed within one day. (ii) Costs and throughput: The minimum number of reads is 100.000, and therefore, many samples, even hundreds, can be analyzed on a benchtop sequencer like the MiSeq or MiniSeq at extremely low costs. In contrast, 3–4 million reads are needed for reliable copy number profiling using WGS libraries and subsequent ichorCNA analysis, which results in a maximal pooling of six samples in one MiSeq run. (iii) Broad patient coverage: Due to the fact that mFAST-SeqS relies on genome-wide SCNAs rather than specific mutations and almost all solid tumors are genetically unstable, it can be applied to a wide range of tumor entities. (iv) mFAST-SeqS requires between 500 pg and 1 ng template DNA as a minimal amount of plasma DNA, while WGS libraries require 5–10 ng. In particular, the speed and cost issues are important features of our approach, which may facilitate its implementation as a routinely used clinical diagnostic tool. Altogether, mFAST-SeqS is a useful tool for untargeted assessment of tumor fraction in MBC and enables fast and cost-effective estimation of both prognosis and treatment response.

In conclusion, in this proof-of-concept trial, we demonstrated a potential of the mFAST-SeqS as a prognostic and monitoring tool in MBC patients. Our results warrant further validation in larger well-defined cohorts of MBC patients prior to its implementation in the clinics.

## 7. Materials and Methods

### 7.1. Patients and Sample Collections

From June 2014–December 2016, 29 female MBC patients were recruited for this prospective exploratory observational follow-up study at the Division of Oncology, Department of Internal Medicine, Medical University of Graz, Austria. The present study was approved by the ethics committee of the Medical University Graz (ethical approval numbers 24-539 ex 11/12 and 21-227 ex 09/10) and written informed consent was obtained from all patients. All experiments were carried out in consideration of the guidelines for good scientific practice as officially required from the Medical University of Graz. Clinical data, as well as histopathological findings, were retrieved from clinical and pathological records. Blood samples were obtained either at first diagnosis of metastases, during several lines of treatment, and/or at every further moment of progression/development of new metastases along with an introduction of a new line of systemic treatment. Samples taken at study recruitment (i.e., the first diagnosis of metastases or introduction of a new line of treatment due to disease progression), were considered as baseline. The therapeutic response was assessed using conventional radiological staging methods.

### 7.2. Preparation of Plasma DNA

Isolation of plasma DNA was performed as previously described [25]. Briefly, whole blood (9 mL) was collected in EDTA vacutainer tubes (BD Biosciences, Heidelberg, Germany) and 0.225 mL of a 10% neutral buffered solution containing formaldehyde (4% weight per volume, Sigma-Aldrich, Vienna, Austria) was added immediately after a blood draw. Plasma was isolated from blood samples by centrifugation at 200 *g* for 10 min with brake and acceleration powers set to zero. The supernatant was then removed and centrifuged at 1600 *g* for an additional 10 min. Plasma was then stored in 2 mL tubes at −70 °C. Plasma DNA was prepared from 1 mL plasma using the QIAamp Circulating Nucleic Acid Kit (Qiagen, Hilden, Germany) according to the manufacturer recommendations. Qubit dsDNA HS Assay Kit (Life Technologies, Vienna, Austria) was used for quantification of plasma DNA.

### 7.3. Modified Fast Aneuploid Screening Test-Sequencing System (mFAST-SeqS)

*LINE1*-amplicon libraries for mFAST-SeqS were prepared as previously described by our group [32]. Briefly, 0.5–2 ng of plasma DNA was amplified with Phusion Hot Start II Polymerase in 5 PCR cycles using target-specific L1 primers. After purification of the PCR products with AMPure Beads (Beckman Coulter, Brea, CA, USA), 10 µL was directly used for the second PCR in which Illumina specific adaptors and indices were added. L1 amplicon libraries were pooled equimolarly and sequenced on an Illumina MiSeq generating 150 bp single reads aiming for at least 100,000 reads. Read counts of each chromosome arm were compared to 18 self-reporting healthy women from a previous study using *z*-score statistics to assess over - and under-representation of *LINE1*-sequences [32]. To get a general measure of aneuploidy, as a surrogate for tumor fraction, normalized read counts per chromosome arm were squared and summed up resulting in a genome-wide *z*-score.

### 7.4. Plasma-Seq

Plasma-Seq, i.e., low-pass whole-genome sequencing (to establish genome-wide copy number alterations) was done as described previously in detail [26]. In brief, shotgun libraries were prepared using the TruSeq DNA LT Sample Preparation Kit (Illumina, San Diego, CA, USA) We used 5–10 ng of input DNA and, due to the nature of ctDNA we omitted, the fragmentation. For selective amplification of the library fragments, we used 20–25 PCR cycles. The libraries were sequenced on an Illumina MiSeq or NextSeq (Illumina, San Diego, CA, USA). Sequencing data were examined with our optimized in-house plasma-Seq script [26,30]. Additionally, data were analyzed with the previously published ichorCNA algorithm to calculate tumor fraction from ultra-low-pass whole-genome sequencing (ULP-WGS) [31].

### 7.5. Enumeration of CTC

For CTC enrichment, 7.5 mL of whole blood was drawn into CellSave tubes (Veridex LLC, Janssen Diagnostics, Rarities, NJ, USA) and CTCs were captured using a size-based microfilter device as described previously [42,43]. Briefly, blood samples (7.5 mL each) were diluted 1:1 with 1× phosphate-buffered saline (PBS) and fixed for 10 min with 1% formalin (Sigma-Aldrich, Vienna, Austria). Following fixation, blood was processed through the microfilter at a constant flow rate of 75 mL/hour using a motorized syringe pump. Afterward, CTC identification and enumeration was done using double immunofluorescence staining, namely pan-cytokeratin (pan-CK) and CD45 antibody. In detail, filters with captured CTCs were placed on a glass microscope slide and blocked at room temperature for 30 min with blocking buffer containing 5% normal goat serum (Life Technologies) and 3% Triton X-100 (Sigma Aldrich). Next, incubation of samples with primary and subsequently secondary antibodies at room temperature, each for 1 h followed. As primary antibodies mouse anti- CD45 (ready-to-use; DAKO, Glostrup, Denmark), and polyclonal rabbit anti-cytokeratin (1:300; DAKO) were used, and as secondary antibodies goat anti-mouse Alexa 594 and goat anti-rabbit Alexa 488 (both 1:100, Life Technologies). Finally, samples were counterstained with 4, 6-diamidino-2-phenylindole (DAPI, Invitrogen) and mounted on coverslips with ProLong Gold Antifade mounting media (Life Technologies). Using a confocal laser-scanning microscope (Zeiss, Oberkochen, Germany), the entire microfilter surface was screened in order to identify CTCs. CTC positivity was defined as a nucleated cell on the filter which showed a CK staining and was +/CD45 negative.

### 7.6. Statistical Analysis

Statistical analyses and plotting of data were performed with GraphPad Prism 6.0 (GraphPad Software, San Diego, CA, USA) and SPSS 23 (SPSS, Inc., Chicago, IL, USA). Continuous variables were reported as medians (25–75th percentile) and categorical variables were summarized as absolute frequencies and percentages. Associations between categorical variables were analyzed with the chi-square test (expected cell counts ≥5) or Fisher’s exact test (expected cell counts <5). Serial *z*-score measurements (*n* = 108, samples obtained after disease progression were omitted from this analysis) were analyzed using a linear mixed-effect model with a random intercept at the patient level to account for the unbalanced *z*-score data (e.g., total number of *z*-score measurements and time points at which *z*-scores were available varied within and between patients) and for the clustered nature of *z*-score measurements within individual patients. The model parameters were estimated using maximum likelihood and an independent variance-covariance structure was assumed for the random effects. The correlation analysis of baseline markers was performed using the nonparametric Spearman correlation. The median follow-up time was estimated using the reverse Kaplan–Meier method [44]. Overall survival (OS) was calculated from the date of blood sampling to the date of patients’ deaths from any cause. For progression-free survival (PFS), the interval beginning with the date of blood sampling to the date of clinical progression or death, whichever came first, was used. If the corresponding event was not observed, the censoring date was the last day of follow-up. Survival curves were estimated using the Kaplan–Meier method, and the differences were analyzed using the log-rank test. Multivariate survival analysis was performed using Cox Proportional-Hazards regression. All *p* values were two-sided and considered statistically significant when <0.05.

## 8. Conclusions

In conclusion, this proof of principle study demonstrates the prognostic impact of ctDNA levels detected with mFAST-SeqS as a very fast and cost-effective means to assess the ctDNA fraction without prior knowledge of the genetic landscape of the tumor. Furthermore, mFAST-SeqS-based ctDNA levels provided an early means of measuring treatment response.

## Figures and Tables

**Table 1:** Clinical and pathological characteristics of metastatic breast cancer (MBC) patients (*n* = 29).

Category	Number	%
Total	29	
Age at the time of sampling (years)		
Median and range	56 (50–68)	
Menopausal status		
Premenopausal	15	51.7
Postmenopausal	14	48.3
Histologic type		
IDC/NST	25	86.2
ILC	2	6.9
Mixed type	1	3.4
Other	1	3.4
Tumor grading (at primary diagnosis)		
G1	0	0
G2	12	41.4
G3	16	55.2
Unknown	1	3.4
Tumor size (primary tumor)		
pT1	10	34.5
pT2	9	31
pT3/pT4	4	13.8
Unknown	6	20.7
Lymph node status		
N0	10	34.5
N1–3	12	41.4
Unknown	7	24.1
ER status		
Negative	5	17.2
Positive	24	82.8
PR status		
Negative	9	31
Positive	20	69
Her2 status		
Negative	21	72.4
Positive	8	27.6
Subtype (primary tumor)		
HR+/Her2−	18	62.1
HR−/Her2−	3	10.3
Her2+	8	27.6
Bone metastases		
No	9	31
Yes	20	69
Lung metastases		
No	16	55.2
Yes	13	44.8
Liver metastases		
No	19	65.5
Yes	10	34.5
Other metastases		
No	7	24.1
Yes	22	75.9
Number of metastatic sites		
One	9	31
Multiple	20	69
Number of previous therapy lines for metastatic disease		
0	18	62.1
1	4	13.8
≥2	7	24.1
OS status		
Alive	19	65.5
Dead	10	34.5
PFS status		
No progress	6	20.7
Progress	23	79.3
CTC ≥ 1		
No	12	41.4
Yes	17	58.6
CTC ≥ 5		
No	21	72.4
Yes	8	27.6
*Z*-score ≥ 3		
No	18	65.5
Yes	10	34.5
CTC (≥1) or *z*-score (≥3) positive		
No	10	34.5
Yes	19	65.5

MBC metastatic breast cancer, IDC/NST invasive ductal carcinoma/not otherwise specified, ILC invasive lobular carcinoma, ER estrogen receptor, PR progesterone receptor, HR hormone receptor, OS overall survival, PFS progression-free survival, CTC circulating tumor cells.

**Table 2:** Pairwise correlation matrix of *z*-score, circulating tumor cells (CTC) count, carcinoembryonic antigen (CEA) and CA15-3.

All Samples (*n* = 127)	*z*-Score	CTC Count	CEA	CA15-3
***z*-score**	-	0.02 (*n* = 97)	**0.26** (*n* = 112)	**0.25** (*n* = 112)
**CTC count**	0.02 (*n* = 97)	-	0.01 (*n* = 84)	**0.30** (*n* = 84)
**CEA**	**0.26** (*n* = 112)	0.01 (*n* = 84)	-	**0.41** (*n* = 111)
**CA15-3**	**0.25** (*n* = 112)	**0.30** (*n* = 84)	**0.41** (*n* = 111)	-

Statistically significant Spearman correlation coefficients (r) are highlighted in bold.

**Table 3:** Associations of baseline *z*-score and CTC positivity with clinicopathological characteristics (*n* = 29 patients).

Variable	*z*-Score < 3	*z*-Score > = 3	*p* Value	CTC <5	CTC > = 5	*p* Value
Menopausal status			*1.000*			*0.215*
Premenopausal	10	5		9	6	
Postmenopausal	9	5		12	2	
Histology			*0.592*			*0.304*
IDC/NST	17	8		17	8	
others	2	2		4	0	
Tumor grading			*0.714*			*0.697*
Grade 1/2	8	5		10	3	
Grade 3	11	5		11	5	
Tumor size (pT) ^#^			*0.252*			*0.121*
pT1	7	3		9	1	
pT2	3	6		5	4	
pT3/4	3	1		4	0	
Lymph nodes (pN) *			*1.000*			*1.000*
pN0	5	5		8	2	
pN1-3	7	5		9	3	
ER status			*0.306*			*0.597*
Negative	2	3		3	2	
Positive	17	7		18	6	
PR status			*1.000*			*1.000*
Negative	6	3		7	2	
Positive	13	7		14	6	
HER2 status			*0.675*			*1.000*
Negative	13	8		15	6	
Positive	6	2		6	2	
Subtype (primary tumor)			*0.065*			*1.000*
HR+/Her2−	13	5		13	5	
HR−/Her2−	0	3		2	1	
Her2+	6	2		6	2	
Bone metastases			*1.000*			*0.371*
No	6	3		8	1	
Yes	13	7		13	7	
Lung metastases			*0.270*			*0.406*
No	12	4		13	3	
Yes	7	6		8	5	
Liver metastases			*0.244*			*0.001*
No	14	5		18	1	
Yes	5	5		3	7	

# pT was not available for 6 patients; * pN status was not available for 7 patients.

## Supplementary Materials

The following are available online at https://www.mdpi.com/2072-6694/11/8/1171/s1. Figure S1: Heat maps of serial plasma samples of seven selected patients. Figure S2: Correlation between mFAST-SeqS *z*-scores and ichorCNA-based tumor fractions in selected individual patients. Figure S3: Kaplan–Meier analysis of overall survival and progression-free survival based on CTC status, CEA and CA15-3 levels. Figure S4: Mean estimates of *z*-scores, CTC counts, CEA and CA15-3 levels are shown during treatment of metastatic breast cancer patients using a mixed model. Figure S5: Longitudinal monitoring of *z*-scores for an additional five patients.
