# Supplementary material for: Untargeted Assessment of Tumor Fractions in Plasma for Monitoring and Prognostication from Metastatic Breast Cancer Patients Undergoing Systemic Treatment
Source: Cancers (Basel). 2019 Aug 14;11(8):1171. doi: 10.3390/cancers11081171 (PMC6721524; doi:10.3390/cancers11081171)
Supplement: Supplementary file 1 [file cancers-11-01171-s001.pdf]

Supplemental Materials: Untargeted assessment of tumor fractions in plasma for monitoring and prognostication from metastatic breast cancer patients undergoing systemic treatment

C. Suppan, I. Brcic, V. Tiran, D. H. Mueller, F. Posch, M. Auer, E. Ercan, P. Ulz, R. J. Cote, R. H. Datar, N. Dandachi, E. Heitzer, M. Balic

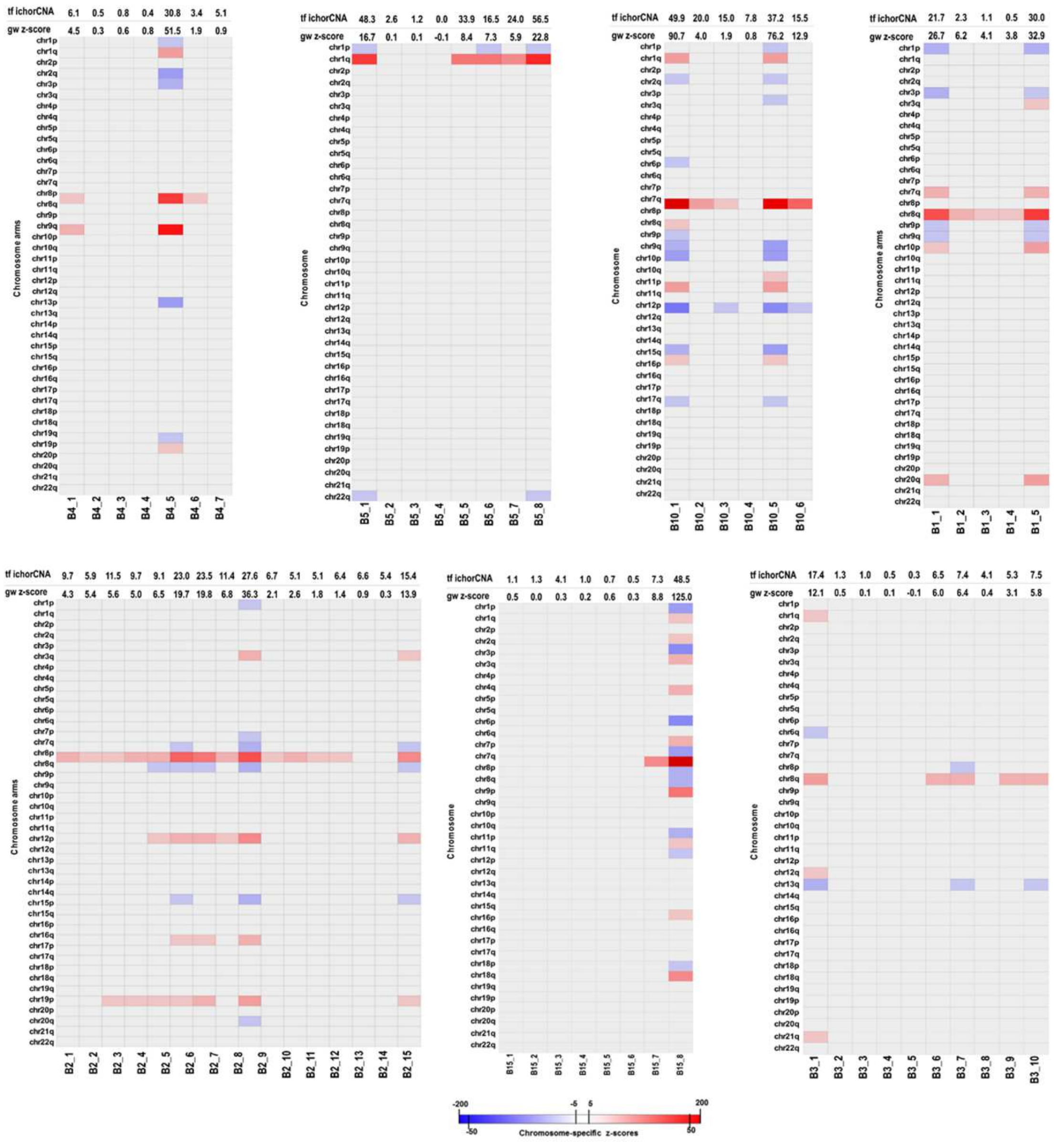

Figure S1. Heat maps of serial plasma samples of seven selected patients showing that chromosome-arm specific CNA are highly consistent within the same patient. Blue bars indicate chromosome-specific z-scores <3, and red bars indicate chromosome-specific z-scores >3. Chr, chromosome. Genomewide (gw) z-scores and tumor fraction (tf) assessed with ichorCNA are shown above the heat map.

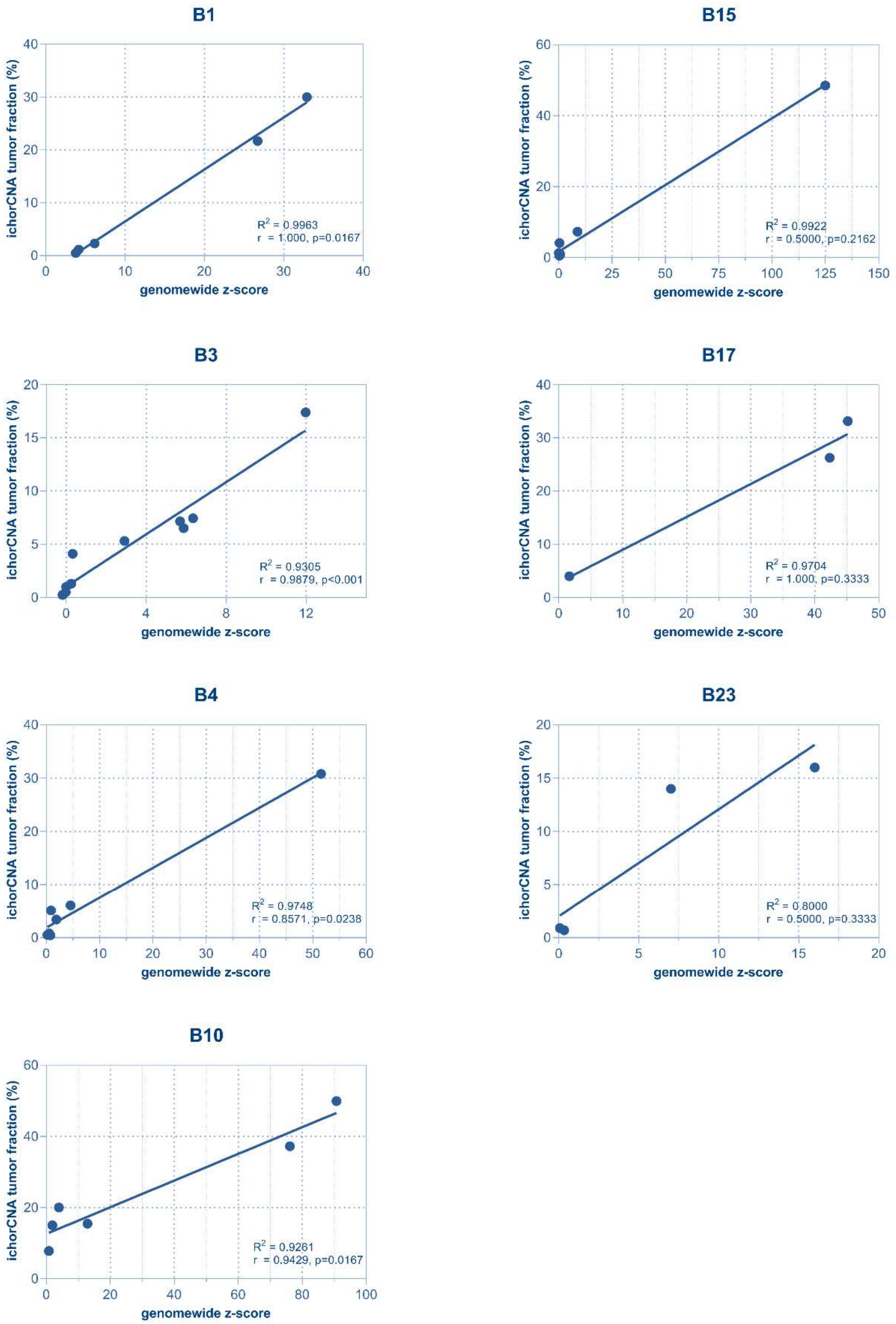

Figure S2. Correlation between mFAST-SeqS z-scores and ichorCNA-based tumor fractions in selected individual patients.

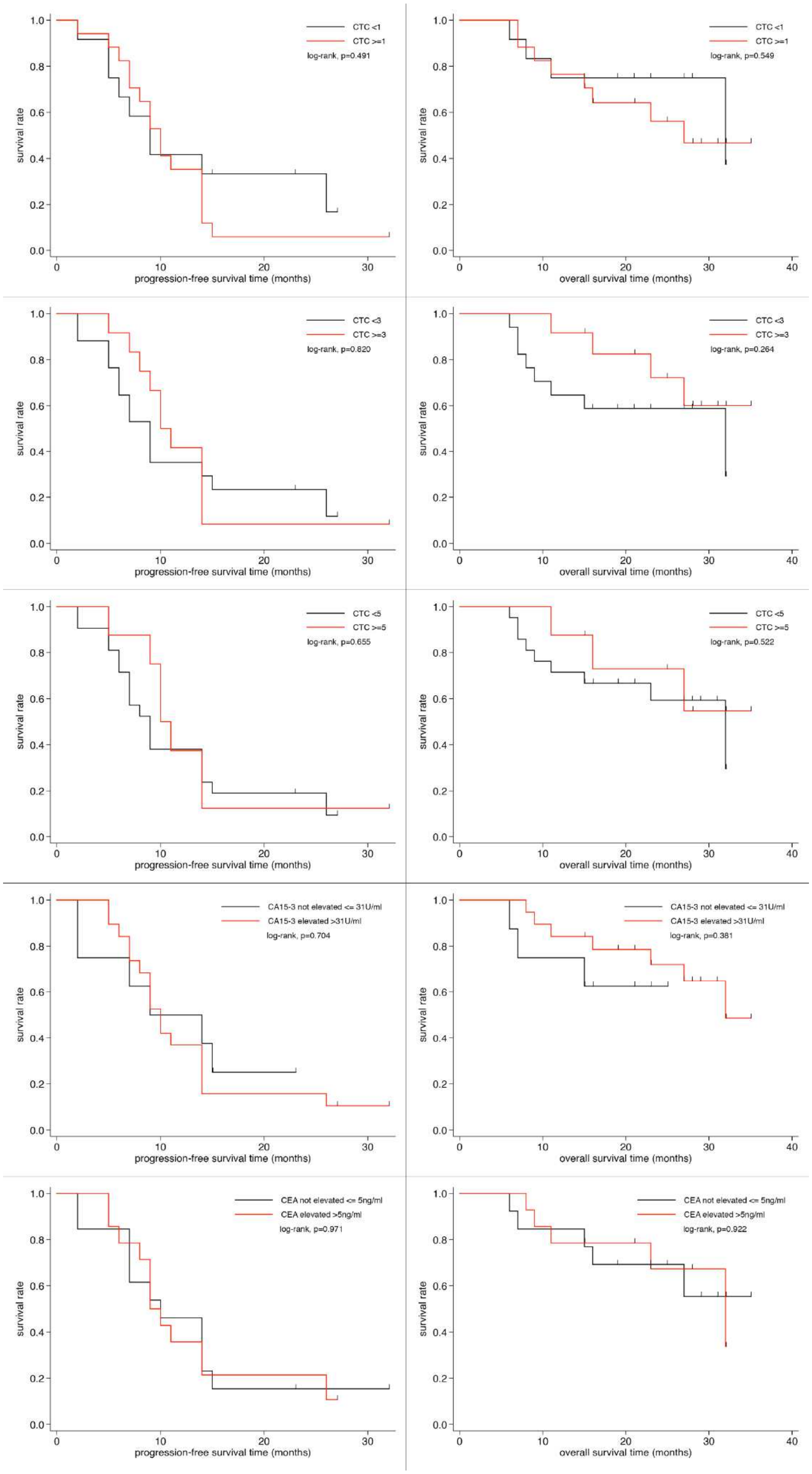

Figure S3. Kaplan Meier analysis of overall survival and progression-free survival based on CTC status, CEA and CA15-3 levels.

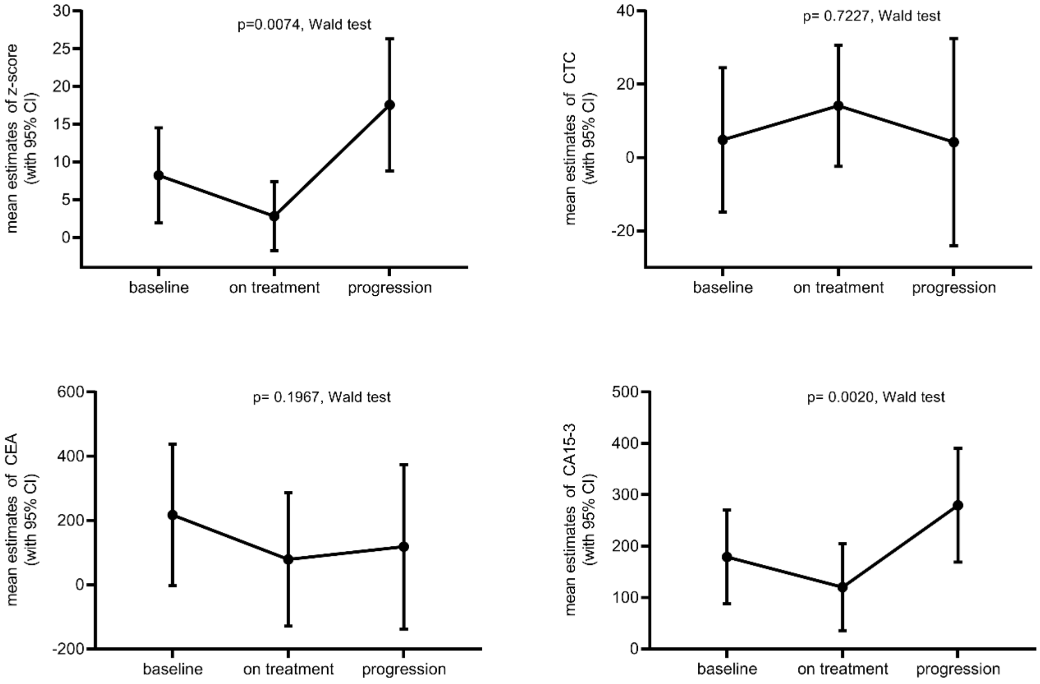

Figure S4. Mean estimates of z-scores, CTC counts, CEA and CA15-3 levels are shown during treatment of metastatic breast cancer patients using a mixed model. Only z-scores and CA15-3 levels were significantly higher at disease progression compared to baseline and treatment. CTC counts and CEA did not significantly differ between the three time points.

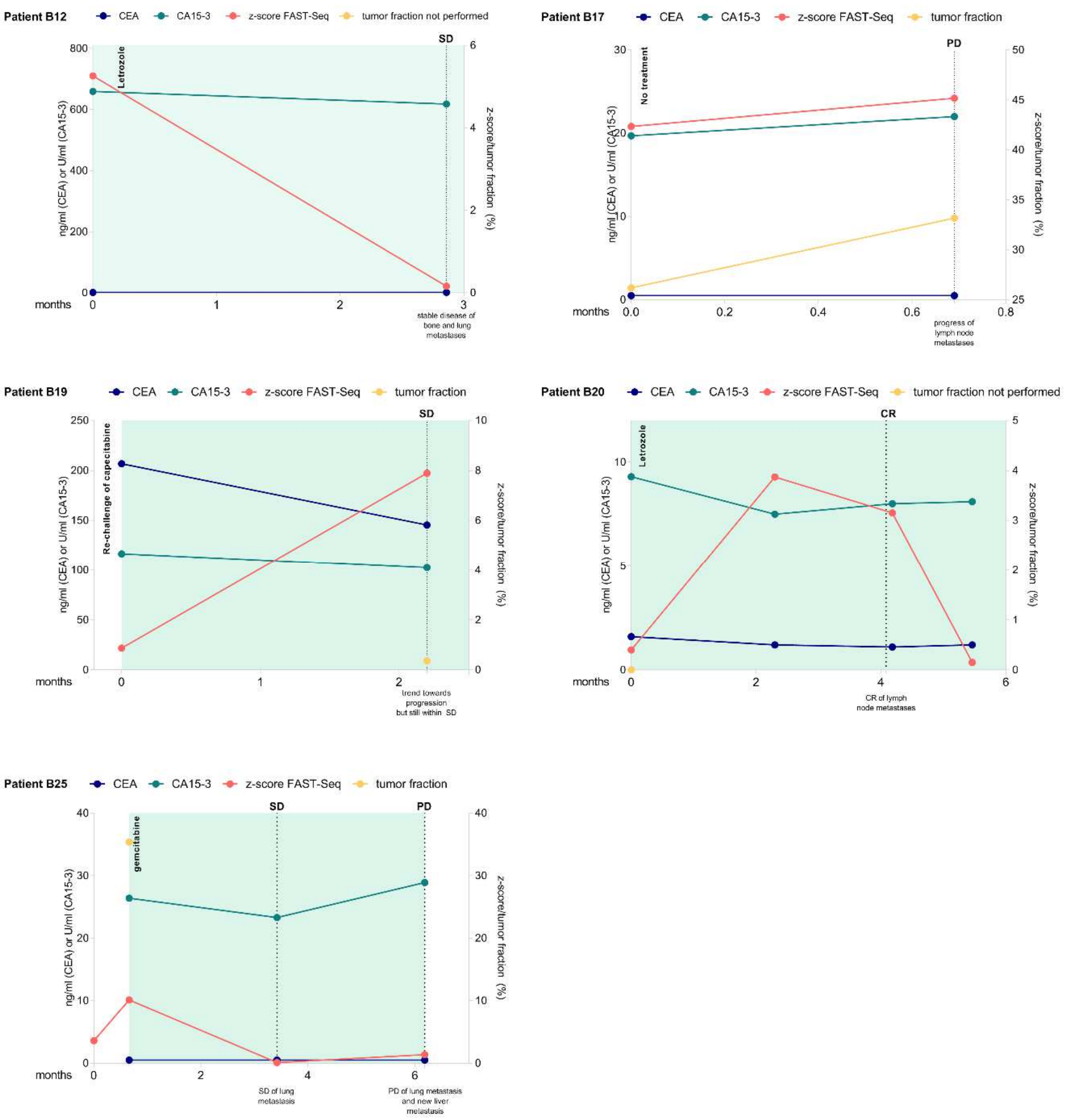

Figure S5. Longitudinal monitoring of z-scores for additional five patients. Multiple measurements of tumor markers CEA and CA15-3 are also shown over time and, if available, ichhorCNA tumor fractions. Therapy regimens are indicated as colored shading and disease status at various times (ascertained on computed tomography or MRI) shown as vertical dotted lines. SD denotes stable disease, PD progressive disease, and CR complete response.
